# Longitudinal maternal hemodynamics from late pregnancy to postpartum in uncomplicated twin pregnancies—A glimpse into long‐term cardiovascular risk?

**DOI:** 10.1111/aogs.70120

**Published:** 2025-12-12

**Authors:** Hannah Friederike Zekert, Anna‐Lena Biermann, Vivien Dütemeyer, Nina Meier, Lena Radomsky, Peter Hillemanns, Constantin von Kaisenberg, Lars Brodowski

**Affiliations:** ^1^ Department of Gynecology and Obstetrics Hannover Medical School Hannover Germany

**Keywords:** cardiovascular function, dichorionic twin pregnancy, maternal hemodynamics, monochorionic twin pregnancy, USCOM

## Abstract

**Introduction:**

Twin pregnancies impose greater cardiovascular demands than singleton gestations, potentially increasing long‐term cardiovascular risk even in the absence of hypertensive disorders. Nevertheless, longitudinal assessments of maternal hemodynamics in uncomplicated twin pregnancies remain limited, and most available studies focus solely on the antenatal period. Chorionicity has been shown to markedly influence cardiovascular adaptation during twin gestation. This study aimed to investigate whether distinct hemodynamic adaptations occur in twin compared with singleton pregnancies during late gestation, 1 day and 6 weeks postpartum.

**Material and Methods:**

In this prospective longitudinal cohort study conducted at Hannover Medical School from 08/24 to 05/2025, 36 women with twin pregnancies (8 monochorionic (MC), 28 dichorionic (DC)) and 37 with singleton pregnancies underwent noninvasive hemodynamic assessment at 34 weeks' gestation, 1 day postpartum, and 6 weeks postpartum.

**Results:**

MC twin pregnancies exhibited significantly higher cardiac output (MC: 7.72 L/min; DC: 5.62 L/min; S: 6.27 L/min; *p* = 0.01) and lower systemic vascular resistance (MC: 958.83 dynes × s/cm^5^; DC: 1206.86 dynes × s/cm^5^; S: 1119.45 dynes × s/cm^5^; *p* = 0.01) during the third trimester, with a similar hemodynamic pattern appearing to persist in the postpartum period. MC twins also showed significant decreases in heart rate (T1: 86.37 bpm; T2: 77.73 bpm; T3: 66.67 bpm; *p* = 0.002), mean arterial pressure (T1: 93.0 mmHg; T2: 85.5 mmHg; T3: 78.0 mmHg; *p* = 0.03), and inotropism postpartum (T1: 1.92 W/m^2^; T2: 1.67 W/m^2^; T3: 1.54 W/m^2^; *p* = 0.04), whereas DC twins demonstrated a trend to higher stroke volume (T1: 69.6 mL; T2: 80.31 mL; T3: 82.63 mL; *p* = 0.01) and gradual increase of vascular resistance (T1: 1206.86 dynes × s/cm^5^; T2:1099.86 dynes × s/cm^5^; T3: 1426.78 dynes × s/cm^5^; *p* = 0.08).

**Conclusions:**

Monochorionic twin pregnancies are characterized by elevated cardiac output and reduced vascular resistance in late pregnancy, with a similar hemodynamic pattern appearing to persist in the postpartum period. This persistent cardiovascular strain may underlie the elevated short‐term cardiovascular risk observed after twin births. Our findings highlight the need for larger longitudinal studies to explore the transition from physiological adaptation to potential cardiovascular maladaptation.

## Graphical abstract

Monochorionic twin pregnancies show higher cardiac output and lower vascular resistance in late pregnancy, a pattern appearing to persist postpartum—A glimpse into long‐term cardiovascular risk?

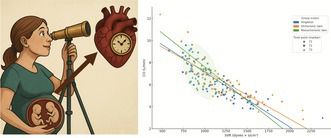

**Abbreviations**
CI: cardiac index
CO: cardiac output
DC: dichorionic
HR: heart rate
INO: Smith–Madigan inotropy index
MAP: mean arterial pressure
MC: monochorionic
PKR: potential‐to‐kinetic‐energy ratio
SV: stroke volume
SVI: stroke volume index
SVR: systemic vascular resistance
SVRI: systemic vascular resistance index
SVV: stroke volume variation

**Key Message**

Monochorionic twin pregnancies show elevated cardiac output and reduced vascular resistance in late pregnancy, with a similar hemodynamic pattern appearing to persist in the postpartum period. This prolonged hemodynamic strain may contribute to increased maternal cardiovascular risk, underscoring the need for targeted postpartum monitoring and longitudinal follow‐up.

## 1 INTRODUCTION

In Germany, the number of twin births is approximately 13 000 per year, accounting for 1.6% of all deliveries.^1^ In recent decades, there has been a consistent rise in the prevalence of these pregnancies, attributable to an increase in the average age of mothers at the time of childbirth, and a rise in the demand for fertility treatments.^2^

Twin gestations have been shown to impose greater physiological demands on the maternal cardiovascular system. This requirement results in a substantially greater hemodynamic load compared with singleton pregnancies. Consequently, maternal cardiovascular adaptations are typically more pronounced in twin gestations, as demonstrated by higher cardiac output (CO), increased stroke volume, and a more extensive plasma volume expansion.^3^ Studies suggest that chorionicity modulates this adaptation. In monochorionic twin pregnancies, it seems that CO increases more during the second trimester compared with dichorionic or singleton gestations.^4^ Due to the greater changes in monochorionic twin pregnancies, these pregnancies may impose a greater cardiovascular burden in comparison with dichorionic and singleton pregnancies.

Twin pregnancies are not only characterized by altered maternal hemodynamics but are also associated with an increased risk of pregnancy‐related complications, including preeclampsia and fetal growth restriction (FGR).^2^, ^3^, ^5^

There is some evidence that even in the absence of hypertensive disorders, women with twin pregnancies exhibit an elevated risk of cardiovascular events during the first year after delivery, in comparison to those with uncomplicated singleton pregnancies.^6^

However, the majority of preceding epidemiological studies have documented a comparable postpartum risk of cardiovascular incidents in women with multiple and singleton pregnancies.^7^, ^8^, ^9^, ^10^, ^11^, ^12^ Some studies even describe a lower risk of postpartum cardiovascular morbidity in multiple pregnancies.^10^, ^12^ The underlying mechanisms remain to be elucidated. It is conceivable that the pathophysiology of preeclampsia in multiple pregnancies may be more pregnancy‐specific and less attributable to preexisting cardiovascular risk factors. Conversely, multiple pregnancies resulting in live births may occur more frequently among women who are already healthier prior to pregnancy or have superior access to medical care.

However, the evidence regarding the long‐term cardiovascular risk of twin pregnancies appears unsatisfactory. To date, a small number of studies have been conducted to investigate short‐term cardiovascular morbidity and mortality in twin pregnancies postpartum. A number of studies have examined maternal hemodynamic adaptation over the course of pregnancy; the majority of these have focused on the antenatal period.^13^, ^14^, ^15^ The available data on maternal cardiovascular recovery and adaptation in the immediate postpartum and early puerperal periods remains limited.^16^

Therefore, the objective of this study was to determine whether distinct hemodynamic adaptations can be observed in uncomplicated twin versus singleton pregnancies during the third trimester, immediately postpartum, and 6 weeks after delivery and if the chorionicity has an impact. The hypothesis is that twin pregnancies without hypertensive disorders have a different hemodynamic profile compared with singletons, which may provide information on the risk of long‐term cardiovascular complications.

## 2 MATERIAL AND METHODS

This prospective longitudinal cohort study was conducted at the Department of Obstetrics and Gynecology, Hannover Medical School, Hannover, Germany between August 2024 and May 2025. Women with uncomplicated singleton and twin pregnancies were recruited. The exclusion criteria were language barriers, chronic hypertension, or complicated pregnancies. Complicated pregnancies were defined as the development of either twin‐to‐twin transfusion syndrome (TTTS), twin anemia‐polycythemia sequence (TAPS), twin reversed arterial perfusion sequence (TRAPS), selective intrauterine growth restriction (sIUGR), FGR, pregnancy‐induced hypertension (PIH), preeclampsia (PE), or HELLP syndrome.^17^, ^18^ The study population comprised 36 patients with twin pregnancies (8 monochorionic diamniotic and 28 dichorionic diamniotic twin pregnancies), and 37 patients with singleton pregnancies, who served as the reference group. All patients were requested to undergo a hemodynamic evaluation on three occasions: at 34 weeks of gestation, 1 day postpartum, and 6 weeks postpartum.

The hemodynamic assessment was conducted by a single operator using an Ultrasonic Cardiac Output Monitor (USCOM®) (USCOM Ltd., Coffs Harbor, Australia). USCOM employs continuous‐wave Doppler as a noninvasive ultrasonic technique to ascertain various cardiovascular parameters.^19^ The ultrasound probe is positioned at the suprasternal notch to measure transaortic blood flow. The patients' height and weight were obtained to determine the aortic outflow tract diameter, as well as the body surface area (BSA) and body mass index (BMI) to calculate indices. Blood pressure was measured using a brachial artery automated machine (OMRON, Model M500 HEM‐7321‐D) to calculate obtained variables.

The evaluated parameters were as follows: heart rate (HR), stroke volume (SV), stroke volume index (SVI), CO, cardiac index (CI), mean arterial pressure (MAP), systemic vascular resistance (SVR), systemic vascular resistance index (SVRI), stroke volume variation (SVV), Smith–Madigan inotropy index (INO), and potential‐to‐kinetic‐energy ratio (PKR). The selected parameters encompass the complete hemodynamic condition, incorporating preload, afterload, and inotropism. The calculation of INO and PKR is based on the evaluation of potential and kinetic energy. Three consecutive USCOM measurements were taken with the patients in a left lateral recumbent position, and two consecutive similar Doppler profiles (cardiac cycles) with the highest and sharpest peak, the least amount of interference, a sharp ascendance and descendance, a clear systolic beginning and end, and a well‐defined image base were included in the analysis. It is important to note that all measurements were taken using the flow trace mode. In conclusion, 89.4% of the USCOM measurements obtained were deemed suitable for analysis.

Maternal, obstetrical, and neonatal characteristics and outcomes were recorded. The measurement of the placental weight was taken. In the case of dichorionic twins, the total weight of both placentas was noted.

Before the data were collected, the ethics committee was asked to evaluate the study (No. 11368_BO_K_2024). All methods were carried out in accordance with relevant guidelines and regulations. Written consent was obtained from all participants.

Statistical analyses were performed using GraphPad Prism 9 (GraphPad Software, La Jolla, CA). The distribution of each continuous variable was assessed separately for every time point using the Shapiro–Wilk normality test. As most variables were nonnormally distributed and sample sizes—particularly in the monochorionic twin group—were small, continuous data are reported as median and interquartile range (IQR), which provides a robust measure of central tendency under these conditions. Categorical variables are presented as counts and percentages.

For between‐group comparisons (monochorionic twins, dichorionic twins, singletons) at individual time points, the Kruskal–Wallis test was used for nonnormally distributed variables, and one‐way analysis of variance (ANOVA) was applied when normality assumptions were met. For within‐group (longitudinal) comparisons across the three measurement time points (T1: third trimester, T2: 1 day postpartum, T3: 6 weeks postpartum), the Wilcoxon signed‐rank test was used for nonparametric data and the paired *t*‐test for normally distributed variables. CO and systemic vascular resistance (SVR) were defined as the primary outcomes. All other hemodynamic variables were analyzed in an exploratory manner. An a priori power consideration was performed for a one‐way analysis of variance (ANOVA) comparing three groups (monochorionic twins, dichorionic twins, and singletons) with the available sample sizes (*n* = 8, 28, and 37, respectively; total *N* = 73) and a two‐sided significance level of *α* = 0.05. Based on Cohen's conventions, we assumed at least a medium effect size (Cohen's *f* = 0.25) and considered large effects (*f* = 0.40) to be clinically relevant between‐group differences in CO and SVR. Under these assumptions, the design provides adequate power (>80%) to detect large between‐group effects, whereas the power to detect medium or small effects is limited. All other hemodynamic variables were analyzed in an exploratory fashion and the study was not specifically powered for these outcomes. Given the exploratory nature of secondary analyses, no formal correction for multiple comparisons was applied. A two‐sided *p*‐value <0.05 was considered statistically significant.

## 3 RESULTS

### 3.1 Study population

The maternal age and prepregnancy BMI were similar in all cohorts. There were no differences in the rate of cesarean section, incidence of gestational diabetes or parity. However, fertility treatment was significantly more frequent in the dichorionic twin group. Birthweight and gestational age at delivery were significantly lower in the twin cohorts, and lowest in MC twins. Overall placental weight was lowest in singleton and highest in DC twin pregnancies. Notably placental weight displayed no significant difference between singleton and MC twins. Baseline characteristics are described in Table 1.

**TABLE 1:** Descriptive parameters of study population

Characteristic	Monochorionic (MC)	Dichorionic (DC)	Singleton (S)	*P*			
				Overall	MC vs. DC	MC vs. S	DC vs. S
Maternal age (years)	33.00 (32.75–34.25)	33.00 (29.00–35.00)	33.00 (31.00–34.50)	0.66	0.25	0.5	0.51
Prepregnancy BMI (kg/m^2^)	24.69 (21.34–25.32)	24.03 (21.28–28.64)	23.70 (20.86–27.67)	0.92	0.68	0.85	0.81
Nulliparous	5/8 (63%)	16/27 (59%)	21/37 (57%)	0.84	0.94	0.77	0.57
Fertility treatment	2/5 (40%)	9/22 (41%)	2/19 (11%)	0.08	0.97	0.12	**0.03**
Cesarean section	5/8 (63%)	12/26 (46%)	19/35 (54%)	0.68	0.42	0.67	0.53
Gestational diabetes	0/6 (0%)	8/23 (35%)	2/32 (6%)	0.55	0.09	0.58	0.30
Gestational age at delivery (days)	256.00 (252.75–259.25)	260.50 (259.00–262.75)	275.00 (271.50–282.50)	**<0.001**	**0.02**	**<0.001**	**<0.001**
Birth weight (g)	2458.75 (2228.13–2569.38)	2637.50 (2464.38–2895.00)	3385.00 (3272.50–3705.00)	**<0.001**	**0.03**	**<0.001**	**<0.001**
Birth‐weight centile (%)	13.00 (11.00–21.00)	22.00 (13.00–31.00)	49.00 (28.00–78.00)	**<0.001**	**0.04**	**<0.001**	**<0.001**
Placental weight (g)	770.00	1010.00 (942.50–1112.50)	600 (512.00–705.00)	**<0.001**	/	/	**<0.001**

*Note:* Data are given as median (interquartile range) or *n* (%). *P* < 0.05: significant difference.

Abbreviation: BMI, body mass index.

### 3.2 Changes in maternal hemodynamics in twin pregnancies

In MC twin pregnancies, HR decreased significantly from the third trimester to 6 weeks postpartum (86.37 vs. 66.67 bpm, *p* = 0.03). As SV remained stable, this reduction in HR led to a corresponding decrease in CO (7.72 vs. 6.84 L/min, *p* = 0.03). Cardiac index declined numerically but not significantly (3.39 vs. 3.30 L/min/m^2^). MAP was lower postpartum (93.00 vs. 78.00 mmHg, *p* = 0.03), and INO showed a small but statistically significant decrease shortly after delivery (1.92 vs. 1.67 W/m^2^, *p* = 0.04). SVV showed a borderline increase 1 day postpartum (2.21 vs. 5.68%, *p* = 0.05) and a nonsignificant decrease thereafter (5.68 vs. 2.33%, *p* = 0.38). SV, SVI, SVR, SVRI, and PKR remained stable across time points.

In DC pregnancies, HR decreased mainly within the first days postpartum (80.13 vs. 73.62 bpm, *p* = 0.02), whereas SV and SVI increased significantly (69.60 vs. 80.31 mL, *p* < 0.01; 35.72 vs. 41.10 mL/m^2^, *p* < 0.01). There was no significant change in CO and CI between the measured timepoints. SVR and SVRI decreased significantly 1 day after delivery (1206.86 vs. 1099.86 dynes × s/cm^5^, *p* = 0.04; and 2418.47 vs. 2148.10 dynes × s/cm^5^/m^2^, *p* = 0.03) and showed a nonsignificant numerical increase 6 weeks after birth (1099.86 vs. 1426.78 dynes × s/cm^5^, *p* = 0.17; 2148.10 vs. 2690.43 dynes × s/cm^5^/m^2^, *p* = 0.28). MAP showed the same pattern (91.67 vs. 83.67 mmHg, *p* = 0.02; 83.67 vs. 88.84 mmHg, *p* = 0.39). INO decreased from the third trimester to 6 weeks postpartum (1.49 vs. 1.47 W/m^2^, *p* = 0.03), as did PKR within the first days (32.05 vs. 22.80, *p* < 0.01). SVV did not change significantly. Variables and cardiovascular changes in all three cohorts are shown in Table 2.

**TABLE 2:** Longitudinal changes during pregnancy in maternal hemodynamic parameters in uncomplicated monochorionic and dichorionic twin pregnancies and singleton pregnancies

Parameter	T1: third trimester	T2: 1 day postpartum	T3: 6 weeks postpartum	*P*			
				Overall	T1 vs. T2	T2 vs. T3	T1 vs. T3
*Monochorionic twin*							
HR (bpm)	86.37 (79.53–90.92)	77.73 (72.57–80.89)	66.67 (66.30–69.91)	**0.002**	**0.03**	0.08	**0.03**
SV (mL)	96.47 (90.26–104.75)	89.87 (80.24–100.57)	90.69 (87.44–102.22)	0.82	0.81	1.0	0.56
SVI (mL/m^2^)	47.18 (46.63–54.39)	49.29 (45.78–49.85)	51.70 (44.85–56.94)	0.76	0.94	0.38	0.31
SVV (%)	2.21 (0.61–4.00)	5.68 (3.85–8.92)	2.33 (2.00–8.81)	0.22	**0.05**	0.38	0.44
CO (L/min)	7.72 (7.44–8.44)	6.89 (6.28–7.31)	6.84 (5.53–6.96)	**0.006**	0.08	0.22	**0.03**
CI (L/min/m^2^)	3.39 (3.71–4.60)	3.78 (3.44–3.89)	3.30 (3.08–3.85)	**<0.001**	0.21	0.35	0.063
MAP (mmHg)	93.00 (89.67–98.84)	85.50 (76.58–93.42)	78.00 (75.67–89.50)	0.17	0.08	0.38	**0.03**
SVR (dynes × s/cm^5^)	958.83 (853.06–974.32)	1008.32 (941.95–1063.92)	1052.82 (928.94–1141.77)	0.21	0.38	0.47	0.44
SVRI (dynes × s/cm^5^/m^2^)	1873.55 (1642.76–1957.29)	1894.15 (1772.70–2019.15)	1894.15 (1830.93–1996.29)	0.84	0.69	1.0	0.84
INO (W/m^2^)	1.92 (1.84–2.17)	1.67 (1.61–1.80)	1.54 (1.46–2.06)	0.16	**0.04**	1.0	0.16
PKR	17.55 (16.48–22.20)	21.24 (16.08–26.10)	20.40 (19.42–22.50)	0.83	0.69	0.58	0.84
*Dichorionic twin*							
HR (bpm)	80.13 (76.94–83.40)	73.62 (66.24–79.79)	69.99 (63.52–74.07)	**0.002**	**0.02**	0.42	**0.01**
SV (mL)	69.60 (64.08–90.38)	80.31 (71.69–94.37)	82.63 (66.29–92.00)	0.42	**0.01**	0.72	**0.01**
SVI (mL/m^2^)	35.72 (32.51–44.17)	41.10 (35.86–51.18)	41.43 (33.68–48.06)	0.24	**0.003**	0.42	**0.001**
SVV (%)	9.71 (3.88–10.72)	6.40 (2.24–10.14)	6.81 (3.84–10.57)	0.59	0.68	0.93	0.79
CO (L/min)	5.62 (5.03–6.95)	6.06 (4.91–7.09)	5.17 (4.55–6.51)	0.32	0.23	0.60	0.74
CI (L/min/m^2^)	2.99 (2.49–3.45)	3.08 (2.43–3.85)	2.78 (2.39–3.47)	0.60	0.12	0.95	0.15
MAP (mmHg)	91.67 (87.67–100.67)	83.67 (76.67–89.00)	88.84 (80.25–96.08)	0.06	**0.02**	0.39	0.44
SVR (dynes × s/cm^5^)	1206.86 (1085.39–1403.57)	1099.86 (925.96–1361.53)	1426.78 (1101.24–1625.33)	0.08	**0.04**	0.17	0.68
SVRI (dynes × s/cm^5^/m^2^)	2418.47 (2037.48–2870.50)	2148.10 (1787.73–2757.94)	2690.43 (2072.25–3322.26)	**<0.001**	**0.03**	0.28	0.74
INO (W/m^2^)	1.49 (1.22–1.67)	1.43 (1.25–1.75)	1.47 (1.29–1.77)	0.86	0.18	0.64	**0.03**
PKR	32.05 (23.35–42.53)	22.80 (20.24–33.39)	25.55 (22.05–51.65)	0.10	**0.002**	0.45	0.31
*Singleton*							
HR (bpm)	77.04 (69.40–83.39)	76.72 (68.68–82.83)	63.49 (61.86–70.63)	**<0.001**	0.37	**0.004**	**<0.001**
SV (mL)	83.13 (73.37–94.08)	89.60 (74.68–102.16)	83.22 (67.78–104.57)	0.51	0.06	0.46	0.66
SVI (mL/m^2^)	41.60 (37.12–48.74)	44.64 (37.62–54.27)	46.35 (39.14–50.03)	0.28	**0.01**	0.77	0.12
SVV (%)	5.25 (2.13–9.80)	5.17 (1.93–9.13)	4.36 (2.00–7.90)	0.86	0.28	0.63	0.15
CO (L/min)	6.27 (5.19–7.24)	6.27 (5.69–7.61)	5.43 (4.75–6.94)	**0.04**	0.64	**0.01**	**0.03**
CI (L/min/m^2^)	2.91 (2.78–3.77)	3.42 (2.71–3.95)	2.86 (2.67–3.63)	0.19	0.31	**0.03**	0.19
MAP (mmHg)	87.67 (81.84–93.50)	77.50 (71.17–81.33)	80.00 (75.00–88.67)	**<0.001**	**<0.001**	0.12	0.12
SVR (dynes × s/cm^5^)	1119.45 (976.85–1260.46)	927.32 (834.99–1077.99)	1241.23 (915.50–1509.66)	**0.01**	**0.008**	**0.01**	0.32
SVRI (dynes × s/cm^5^/m^2^)	2166.20 (1941.99–2573.30)	1896.16 (1585.38–2144.42)	2357.61 (1612.93–2616.74)	**0.02**	**0.002**	**0.03**	0.85
INO (W/m^2^)	1.57 (1.32–1.78)	1.45 (1.25–1.76)	1.54 (1.25–1.67)	0.54	0.19	0.94	0.32
PKR	24.15 (21.26–28.41)	20.61 (16.26–25.89)	25.54 (19.42–37.66)	**0.04**	0.05	**0.03**	0.98

*Note:* Data are given as median (interquartile range). *p* < 0.05: significant difference.

Abbreviations: CI, cardiac index; CO, cardiac output; HR, heart rate; INO, inotropy Index; MAP, mean arterial pressure; PKR, potential‐to‐kinetic‐energy ratio.SV, stroke volume; SVI, stroke volume index; SVR, systemic vascular resistance; SVRI, systemic vascular resistance index; SVV, stroke volume variation;

### 3.3 Longitudinal comparison of hemodynamics in singleton and twin pregnancies

**FIGURE 1:**
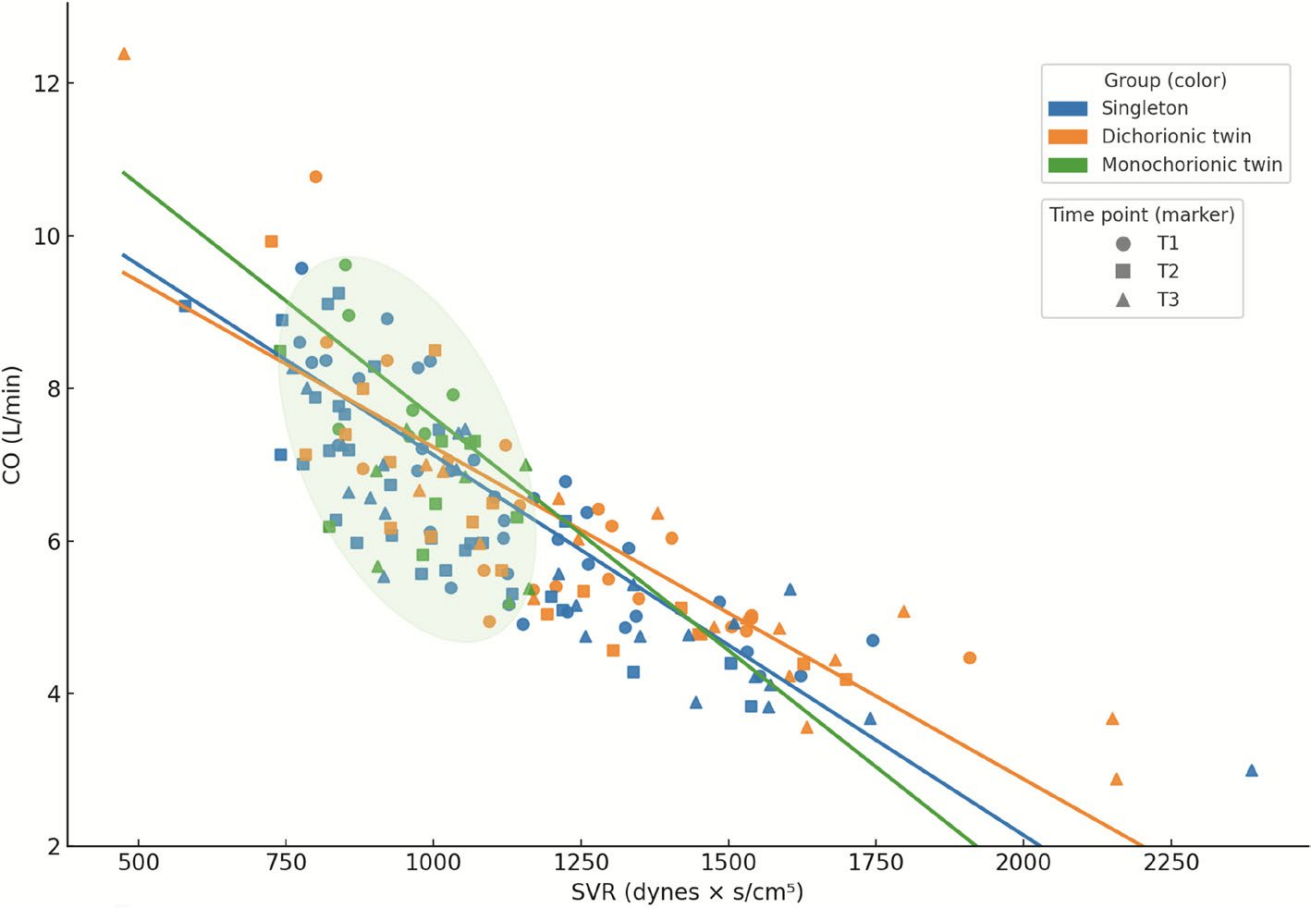
Presentation of all measurement time points in singleton and twin pregnancies, illustrating cardiac output (CO) and systemic vascular resistance (SVR). To explore the relationship between SVR and CO, we performed simple linear regression analyses separately for singleton, dichorionic twin, and monochorionic twin pregnancies. All available measurements from the three time points were pooled within each group to estimate group‐specific regression slopes. Across all three groups, SVR and CO were inversely related. The regression slope was −0.0050 L·min^−1^ per dynes × s/cm^5^ in singleton pregnancies, −0.0044 in dichorionic twins, and −0.0061 in monochorionic twins, indicating a slightly steeper decline of CO with increasing SVR in monochorionic twin pregnancies.

At the third trimester, CO differed significantly across groups, with highest values in MC pregnancies (6.27 L/min in singletons, 7.72 L/min in monochorionic twins and 5.62 L/min in dichorionic twins). Immediately postpartum and at 6 weeks postpartum, CO remained numerically highest in MC twins, although differences were not significant. For SVR, MC twins showed the lowest values at the third trimester and at 6 weeks postpartum, while groups converged immediately postpartum. Significant differences in SVR were observed at third trimester measurement; no difference was observed at 1 day after delivery or 6 weeks postpartum. In summary the descriptive pattern shows higher values for CO and lower values for SVR in MC pregnancies at third trimester and 6 weeks postpartum, whereas values converge immediately postpartum. Simple linear regression analyses demonstrated an inverse descriptive relationship between SVR and CO in all groups, with comparable slopes (singleton: −0.0050; dichorionic: −0.0044; monochorionic: −0.0061 L/min per dynes·cm^5^) (Figure 1). These slopes reflect group‐specific descriptive patterns rather than inferential differences. Regression analyses for each time point are shown in Table S2 and Figures [Link], [Link], [Link].

**FIGURE 2:**
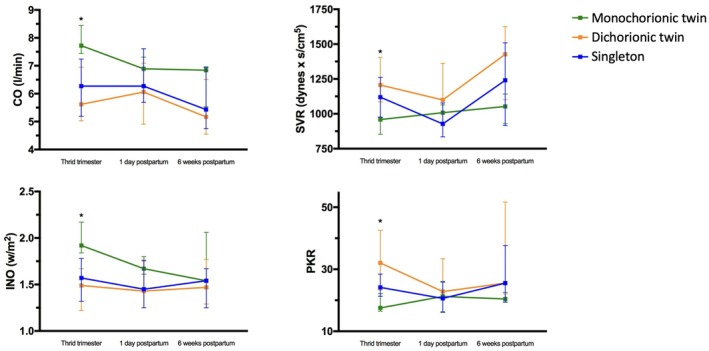
Longitudinal changes in cardiac output (CO), systemic vascular resistance (SVR), potential‐to‐kinetic‐energy ratio (PKR), and inotropy index (INO) in singleton, dichorionic, and monochorionic twin pregnancies. Data were collected at three time points (T1–T3); corresponding sample sizes were: Singleton—35/30/25; dichorionic—21/19/18; monochorionic—7/8/7. Significant within‐group changes are marked (*p* < 0.05).

INO values were significantly highest in monochorionic twins during the third trimester and remained numerically higher postpartum. Dichorionic twins and singletons showed lower and comparable INO values throughout the observation period. PKR values were significantly lower in MC pregnancies during the third trimester, with apparent convergence postpartum. Figure 2 illustrates the longitudinal changes in CO, SVR, INO and PKR from the third trimester to 6 weeks postpartum, highlighting group‐specific differences in cardiovascular adaptation.

## 4 DISCUSSION

This prospective longitudinal study examined maternal hemodynamic changes in singleton, dichorionic, and monochorionic twin pregnancies from the third trimester to 6 weeks postpartum. MC twin pregnancies were characterized by elevated CO and reduced SVR in late pregnancy compared with the other groups. Although postpartum differences were not statistically significant, the general pattern of higher CO and lower SVR in MC twins appeared to persist. Furthermore, MC pregnancies demonstrated a significant decrease in heart rate, MAP, and INO during the postpartum period. In the context of DC twin pregnancies, cardiac adaptation exhibited enhanced stability, manifesting as an immediate surge in stroke volume following delivery and a moderate normalization of MAP during the postpartum phase.

Despite the elevated risk of preeclampsia and increased cardiovascular burden in twin pregnancies, extant literature demonstrates no long‐term increase in cardiovascular morbidity or mortality in twin pregnancies compared with singletons. The majority of these studies focused on the period between six and 10 years after delivery.^7^, ^8^, ^9^, ^10^, ^11^ However, these findings are at odds with those reported by a substantial, contemporary US study encompassing over 36 million births, which observed a higher incidence of heart disease and stroke among patients with twin pregnancies up to 12 months postpartum.^6^ The findings of this study indicate that a period of more than 1 year may be required for the cardiovascular system to return to its prenatal level of function. This is evidenced by the persistent elevated risk of heart disease readmission observed in twins even not afflicted by hypertensive disorders of pregnancy, after the lapse of 1 year following birth.

In the present study, we were able to demonstrate hemodynamic changes primarily in MC twins, which were still detectable 6 weeks after birth. Farsetti et al. previously demonstrated in a longitudinal cohort study that MC twin pregnancies exhibit a pronounced increase in CO in the second trimester, followed by a reduction in cardiac performance in the third trimester—a so‐called “crossover pattern” of maternal hemodynamics.^2^ These alterations are presumably indicative of the restricted cardiac reserve necessary to satisfy the augmented placental demand observed in MC pregnancies. The findings of our present study suggest a persistent trend of elevated CO with concomitant decreased SVR in the third trimester and a decline in cardiac performance postpartum with persistence of elevated CO and decreased SVR in MC twin pregnancies whereas DC and singleton pregnancies display a comparable pattern over the observation time points.

During pregnancy, there is a physiological increase in the volume of both noncirculating and circulating body fluids, which can potentially induce stress on the maternal cardiovascular system.^20^ This is associated with an increase in CO from the second trimester until a plateau at term and with a decrease in peripheral vascular resistance until a nadir in the early third trimester.^21^

It is known that the CO during pregnancy exerts a substantial influence on infant birthweight, with higher CO correlating with larger babies.^22^ Increased CO in pregnancy appears to be essential for adequate placental perfusion, while inefficient vascular adaptation is associated with an increased risk of growth restriction and hypertensive complications.^23^ The observed differences between MC and DC pregnancies, as well as singleton pregnancies, could therefore be indicative of disparate placental development dynamics. Placentas of monochorionic twins do not conform to the principle of allometric metabolic scaling, displayed by comparable placental weight of MC and singleton pregnancies. The increasing fetal‐placental demand must therefore be met early on by increasing placental mass.^24^ This can be achieved by the early increase in CO and decrease in SVR, which is detectable in our study cohort even in the third trimester compared with dichorionic twin and singleton pregnancies.

It is interesting to note that longitudinal observations from the first trimester to term have revealed latent patterns of CO and peripheral vascular resistance during the development of preeclampsia. While early‐onset preeclampsia with FGR is associated with high peripheral vascular resistance and low CO starting in the first trimester,^25^, ^26^ late‐onset preeclampsia without FGR is characterized by circulatory dysfunction with high CO and low SVR at all stages of pregnancy, during delivery, and after birth.^27^ The pattern of late‐onset preeclampsia without FGR describes the hemodynamic profile exhibited by the MC twin pregnancies in our study. Maternal hemodynamic maladaptation with low CO and earlier depletion of the growth and supply reserve in monochorionic placentas, or, conversely, excessive CO may contribute to the increased risk of developing hypertensive disorders in MC pregnancies.^28^, ^29^, ^30^

Most studies have observed that women experiencing twin pregnancies showed altered maternal adaptation during pregnancy in comparison to those experiencing singleton pregnancies. However, these alterations in maternal adaptation have not been shown to persist for a significant period, extending beyond the immediate postpartum phase. The partial normalization of hemodynamics seen in the present cohort 6 weeks postpartum, particularly in DC pregnancies, suggests a gradual reversal of these changes. In contrast, MC twin pregnancies may require pronounced hemodynamic adaptation that extends beyond the pregnancy itself. The persistent trend of elevated CO and reduced vascular resistance in the MC twin group during the postpartum period may partly explain why women with twin pregnancies have an increased risk of postpartum cardiovascular events. The observed reduction in INO and MAP after delivery, as well as the rebound in SVR after 6 weeks, could be interpreted as a sign of the beginning of vascular readjustment. These findings may have the potential to inform future clinical practice, as the persistent elevated CO and reduced vascular resistance in late pregnancy, with a similar hemodynamic pattern appearing to persist in the postpartum period may partly explain why women with twin pregnancies have an increased risk of postpartum cardiovascular events. Nevertheless, the causal link between these hemodynamic alterations and long‐term cardiovascular morbidity remains hypothetical, and further prospective studies with extended follow‐up are needed to substantiate this assumption. We hypothesize that targeted cardiovascular follow‐up after multiple births, particularly in MC pregnancies, may help identify early signs of hemodynamic decompensation during the postpartum readjustment period. Such an approach could potentially reduce the risk of later cardiovascular complications. However, this assumption remains speculative and cannot be generalized to all women with multiple pregnancies. Similarly, while preconception counseling in the context of fertility treatments has shown promise, its relevance to postpartum cardiovascular outcomes requires further study.

The present study is subject to several limitations. The limited sample size, particularly within the MC cohort, constrained the feasibility of conducting subgroup analyses. Our power considerations indicate that the study is sufficiently powered to detect large between‐group differences in the primary hemodynamic outcomes (CO and SVR), but is underpowered to reliably detect small to medium effects. Therefore, nonsignificant results, especially for subgroup analyses and exploratory secondary variables, should be interpreted with caution and cannot be taken as evidence of the absence of more subtle hemodynamic differences. As this was a single‐center investigation, selection bias cannot be excluded, and the generalizability of the findings may be limited. Although strict inclusion criteria were applied, residual confounding by maternal characteristics such as lifestyle factors, comorbidities, or medication use cannot be ruled out. Finally, the follow‐up period was restricted to 6 weeks postpartum, and therefore, the long‐term implications of the observed hemodynamic changes remain speculative. Baseline characteristics differed across the three groups, including gestational age, birthweight, and the proportion of pregnancies conceived following fertility treatment. These factors may influence maternal cardiovascular adaptation and therefore represent potential confounders. Due to the limited sample size, particularly in the monochorionic twin group, multivariable adjustment was not feasible without risking model instability. As a consequence, residual confounding cannot be excluded, and the observed between‐group differences should be interpreted with caution. Echocardiographic parameters that would facilitate a structural assessment of cardiac morphology were not collected. Future studies should validate the patterns identified here in larger cohorts and supplement them by combining USCOM measurements, echocardiography, and placental biomarkers. Furthermore, prospective follow‐up beyond the first postpartum year would be required to conclusively assess the clinical relevance of these hemodynamic changes for subsequent cardiovascular risk.

## 5 CONCLUSION

Monochorionic twin pregnancies exhibit a late‐pregnancy profile of elevated CO and reduced vascular resistance, and postpartum data suggest a possible continuation of this pattern. This hemodynamic variation compared with singleton and dichorionic twin pregnancies may play a key role in the increased short‐term cardiovascular risk after birth. Structured cardiovascular follow‐up and further longitudinal studies are needed to better understand the transition from transient adaptation to pathological stress.

## AUTHOR CONTRIBUTIONS

Anna‐Lena Biermann and Lars Brodowski: design of study, applying for ethical approval, data preparation, statistical analysis, clinical interpretation, writing, and amending manuscript, submitting for publication. Hannah Friederike Zekert: data curation and preparation, statistical analysis, writing, and amending manuscript. Vivien Dütemeyer and Lena Radomsky: design of study, clinical interpretation, amending manuscript. Constantin von Kaisenberg, Peter Hillemanns, and Nina Meier: clinical interpretation, supervision, amending manuscript. All authors contributed to the interpretation of the data and read and approved the final version of the article for publication.

## FUNDING INFORMATION

This study was supported by KlinStrucMed Program and Hannover Medical School, funded by ‘Familie Felling Stiftung’.

## CONFLICT OF INTEREST STATEMENT

The authors declare that they have no competing interests.

## ETHICS STATEMENT

The study was approved on April 23, 2024, by the ethics committee of Hanover Medical School (No. 11368_BO_K_2024).

## Supporting information


**Table S1.** Linear regression of systemic vascular resistance (SVR) predicting cardiac output (CO) across pregnancy groups. All available measurements from T1, T2, and T3 were pooled within each group. The negative slopes indicate an inverse relationship between SVR and CO across all pregnancy types. Monochorionic twins show a steeper decline (more negative slope), but also substantially greater variability (lower *R* ^2^), consistent with the smaller sample size. *R* ^2^ values are provided for completeness but were not displayed in the main figure, in accordance with the journal’s guideline to minimize visual clutter. Model: CO = *β*₀ + *β*₁·SVR.
**Table S2.** Linear regression of systemic vascular resistance (SVR) predicting cardiac output (CO) across pregnancy groups separated by time points T1 to T3. Model: CO = *β*₀ + *β*₁·SVR.

**Figure S1.** Presentation of all measurement of time point T1 in singleton and twin pregnancies, illustrating cardiac output (CO) and systemic vascular resistance (SVR). To explore the relationship between SVR and CO, we performed simple linear regression analyses separately for singleton, dichorionic twin, and monochorionic twin pregnancies.

**Figure S2.** Presentation of all measurement of time point T2 in singleton and twin pregnancies, illustrating cardiac output (CO) and systemic vascular resistance (SVR). To explore the relationship between SVR and CO, we performed simple linear regression analyses separately for singleton, dichorionic twin, and monochorionic twin pregnancies.

**Figure S3.** Presentation of all measurement of time point T3 in singleton and twin pregnancies, illustrating cardiac output (CO) and systemic vascular resistance (SVR). To explore the relationship between SVR and CO, we performed simple linear regression analyses separately for singleton, dichorionic twin, and monochorionic twin pregnancies.

## Data Availability

The data that support the findings of this study are available from the corresponding author upon reasonable request.
