# Supplementary material for: Longitudinal maternal hemodynamics from late pregnancy to postpartum in uncomplicated twin pregnancies—A glimpse into long‐term cardiovascular risk?
Source: Acta Obstet Gynecol Scand. 2025 Dec 12;105(2):288–97. doi: 10.1111/aogs.70120 (PMC12856702; doi:10.1111/aogs.70120)
Supplement: Supplementary file 1 — Table S1. Linear regression of systemic vascular resistance (SVR) predicting cardiac output (CO) across pregnancy groups. All available measurements from T1, T2, and T3 were pooled within each group. The negative slopes indicate an inverse relationship between SVR and CO across all pregnancy types. Monochorionic twins show a steeper decline (more negative slope), but also substantially greater variability (lower R 2), consistent with the smaller sample size. R 2 values are provided for completeness but were not displayed in the main figure, in accordance with the journal’s guideline to minimize visual clutter. Model: CO = β₀ + β₁·SVR. Table S2. Linear regression of systemic vascular resistance (SVR) predicting cardiac output (CO) across pregnancy groups separated by time points T1 to T3. Model: CO = β₀ + β₁·SVR. [file AOGS-105-288-s001.docx]

**Supporting Information Table S1:** Linear regression of systemic vascular resistance (SVR) predicting cardiac output (CO) across pregnancy groups

| Group | Slope β₁ (L·min⁻¹ per dyn·s·cm⁵) | Intercept β₀ (L/min) | R² | n |
| --- | --- | --- | --- | --- |
| Singleton | –0.00418 | 10.94 | 0.694 | 90 |
| Dichorionic twin | –0.00418 | 11.26 | 0.697 | 58 |
| Monochorionic twin | –0.00490 | 11.90 | 0.267 | 22 |

**Supporting Information Table S2:** Linear regression of systemic vascular resistance (SVR) predicting cardiac output (CO) across pregnancy groups separated by time points T1 to T3.

| Linear regression of SVR predicting CO at Time point T1 | | | | |
| --- | --- | --- | --- | --- |
| Group | Slope β₁ (L·min⁻¹ per dyn·s·cm⁵) | Intercept β₀ (L/min) | R² | n |
| Singleton | –0.00498 | 12.11 | 0.714 | 35 |
| Dichorionic twin | –0.00435 | 11.58 | 0.610 | 21 |
| Monochorionic twin | –0.00611 | 13.72 | 0.288 | 7 |

| Linear regression of SVR predicting CO at Time point T2 | | | | |
| --- | --- | --- | --- | --- |
| Group | Slope β₁ | Intercept β₀ | R² | n |
| Singleton | –0.00546 | 11.98 | 0.696 | 30 |
| Dichorionic twin | –0.00467 | 11.49 | 0.733 | 19 |
| Monochorionic twin | –0.00237 | 9.22 | 0.135 | 8 |

| Linear regression of SVR predicting CO at Time point T3 | | | | |
| --- | --- | --- | --- | --- |
| Group | Slope β₁ | Intercept β₀ | R² | n |
| Singleton | –0.00339 | 9.87 | 0.763 | 25 |
| Dichorionic twin | –0.00426 | 11.60 | 0.767 | 18 |
| Monochorionic twin | –0.00273 | 9.18 | 0.121 | 7 |
