# Supplementary figures and images for: Longitudinal maternal hemodynamics from late pregnancy to postpartum in uncomplicated twin pregnancies—A glimpse into long‐term cardiovascular risk?
Source: Acta Obstet Gynecol Scand. 2025 Dec 12;105(2):288–97. doi: 10.1111/aogs.70120 (PMC12856702; doi:10.1111/aogs.70120)

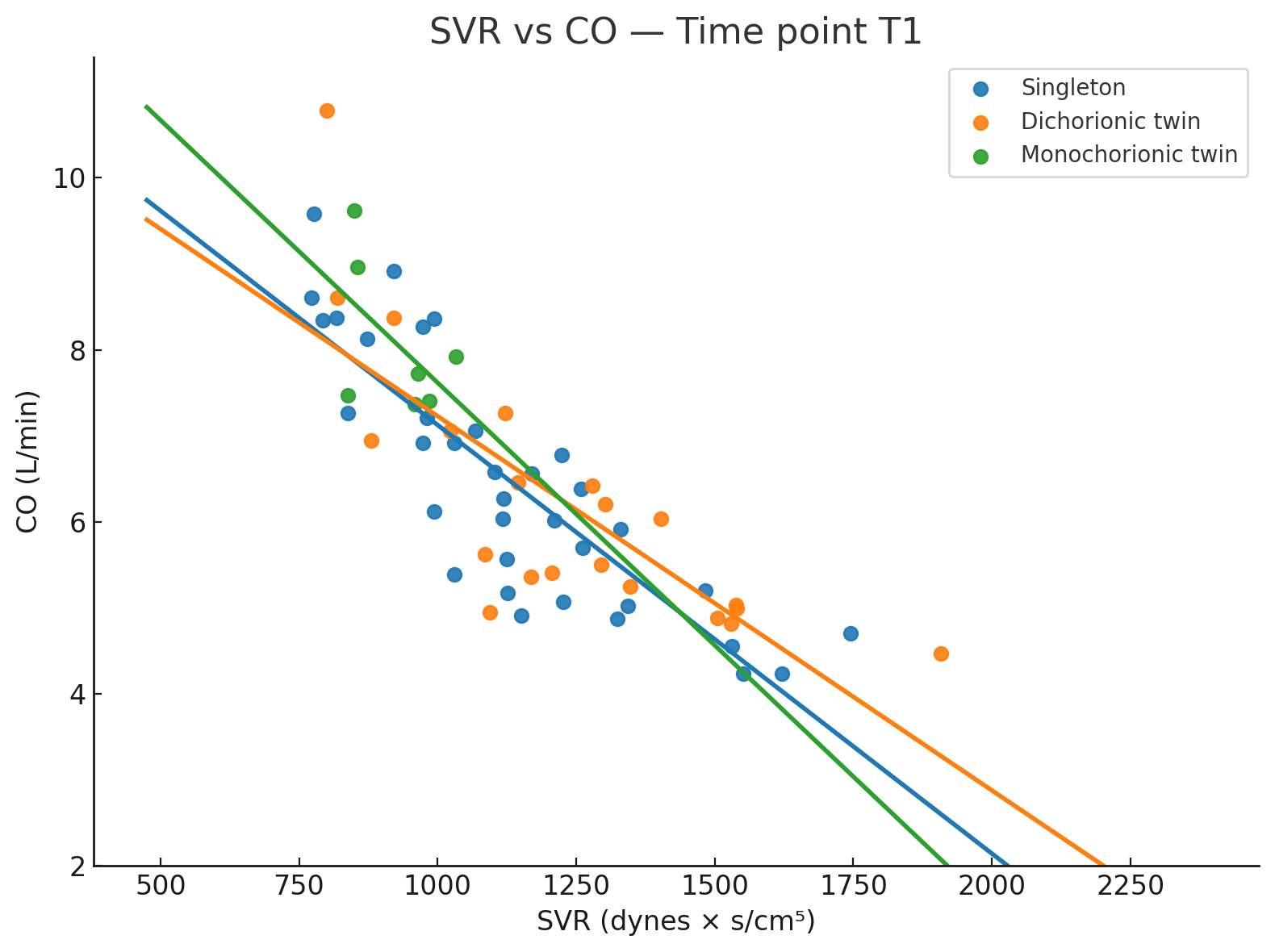

Supplement: Supplementary file 2 — Figure S1. Presentation of all measurement of time point T1 in singleton and twin pregnancies, illustrating cardiac output (CO) and systemic vascular resistance (SVR). To explore the relationship between SVR and CO, we performed simple linear regression analyses separately for singleton, dichorionic twin, and monochorionic twin pregnancies. [file AOGS-105-288-s004.png]

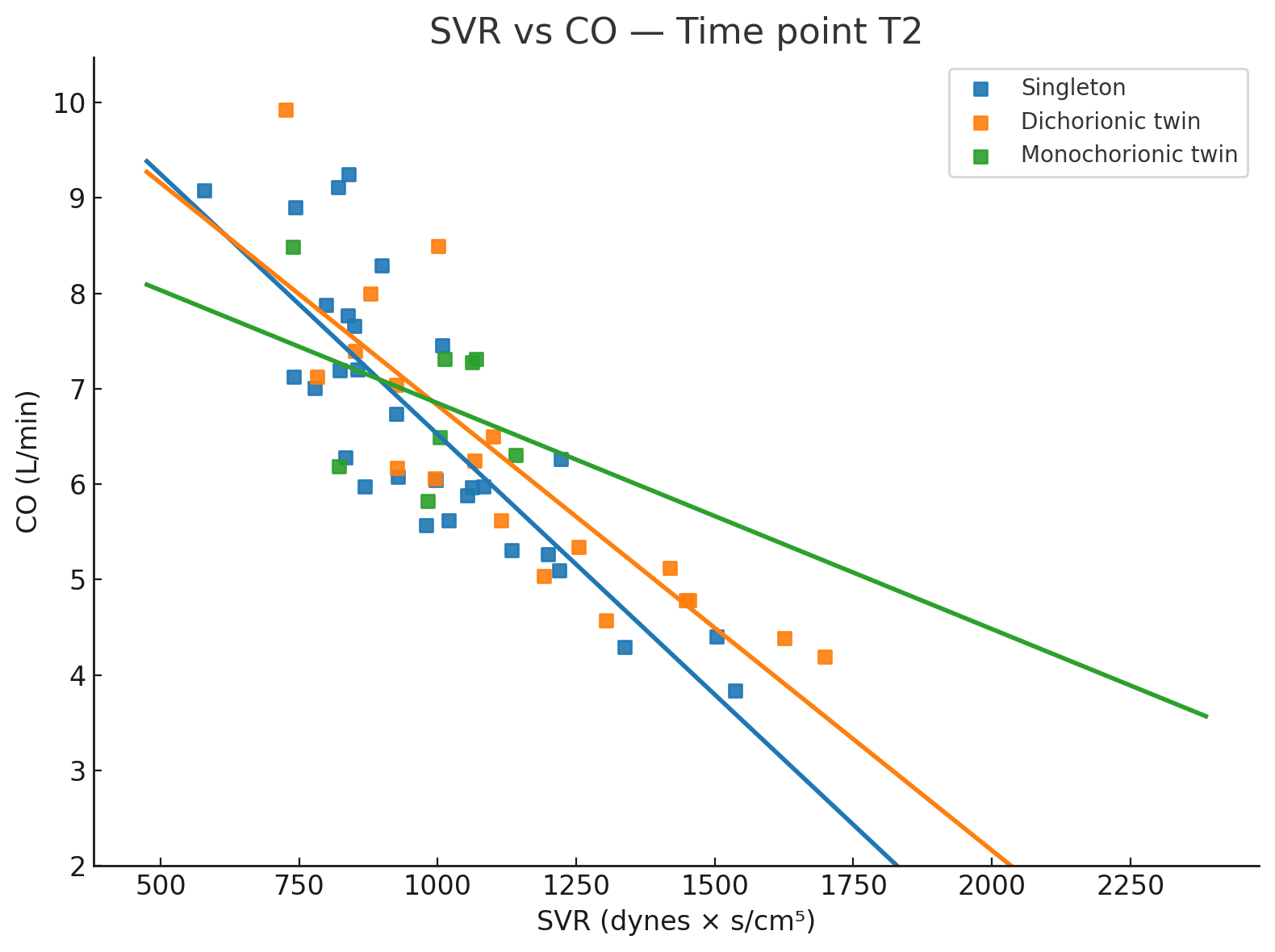

Supplement: Supplementary file 3 — Figure S2. Presentation of all measurement of time point T2 in singleton and twin pregnancies, illustrating cardiac output (CO) and systemic vascular resistance (SVR). To explore the relationship between SVR and CO, we performed simple linear regression analyses separately for singleton, dichorionic twin, and monochorionic twin pregnancies. [file AOGS-105-288-s003.png]

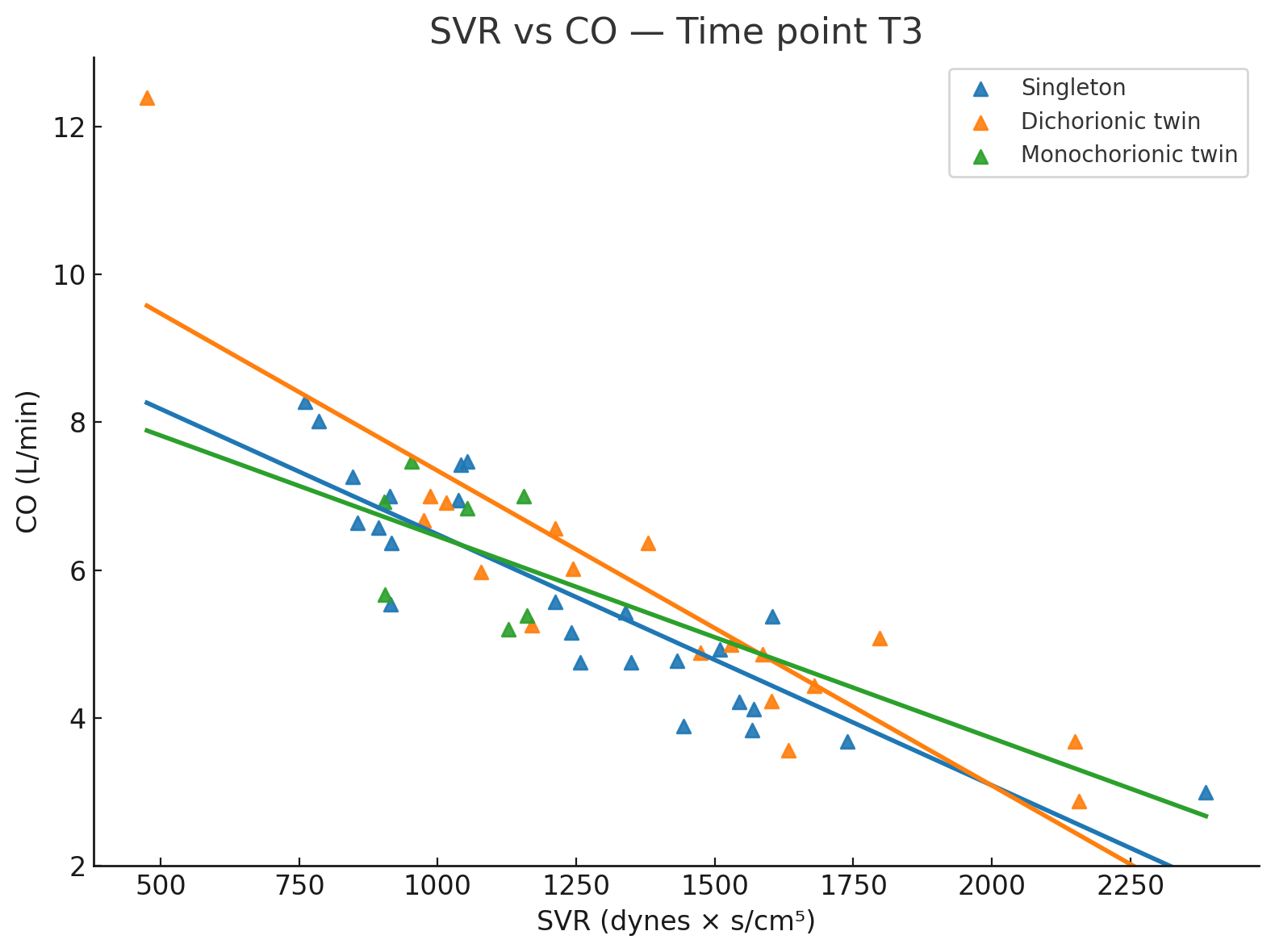

Supplement: Supplementary file 4 — Figure S3. Presentation of all measurement of time point T3 in singleton and twin pregnancies, illustrating cardiac output (CO) and systemic vascular resistance (SVR). To explore the relationship between SVR and CO, we performed simple linear regression analyses separately for singleton, dichorionic twin, and monochorionic twin pregnancies. [file AOGS-105-288-s002.png]
